# Case report: Bilateral globus pallidus lesions and delayed progressive leukoencephalopathy in COVID-19: Effects of hypoxia alone or combination of hypoxia and inflammation?

**DOI:** 10.3389/fneur.2022.1084831

**Published:** 2023-01-09

**Authors:** Ahmad A. Ballout, Michael Kolesnik, Yuna Choi, Marc S. Ayoub, Asaff Harel, Souhel Najjar

**Affiliations:** Department of Neurology, Northwell Health, Donald and Barbara Zucker School of Medicine at Hofstra/Northwell, Hempstead, NY, United States

**Keywords:** COVID-19, leukoencephalopathy, globus pallidus, SARS-CoV-2, hypoxia

## Abstract

**Background:**

The globus pallidus is a highly mitochondria-rich metabolic structure that is particularly sensitive to metabolic disturbances and hypoxia. Symmetric lesions of globus pallidus and delayed diffuse leukoencephalopathy were documented in toxic–metabolic disorders, hypoxia, a neurodegenerative disorder, and mitochondrial encephalopathies. Similar changes are also reported in individuals with active COVID-19 infections with associated hypoxia or critical illness.

**Case information:**

We describe a patient with post–COVID-19 infection who presented with rapid cognitive and neurological decline associated with similar neuroimaging structural changes but without toxic–metabolic changes or hypoxia. Despite multiple non-inflammatory cerebrospinal fluid studies, mechanisms involving post–COVID-19 inflammation and immune dysregulation are suspected, given the unexplained continued decline in the neurological status, lack of concurrent hypoxia or antecedent respiratory difficulties, and after a reasonable exclusion of alternative etiologies. Hypermetabolism of both anteromedial temporal structures and diffuse hypometabolism predominantly in the frontal region on PET scan provided indirect support for possible inflammatory mechanisms after reasonable exclusion of alternative etiologies, such as direct CNS infection, among others. The patient's neurological impairment improved substantially after treatment with pulse steroids, plasmapheresis, and rituximab.

**Conclusion:**

To the best of our knowledge, this is the first report of post–COVID-19 with bilateral symmetric contrast-enhancing necrotic lesions of globus pallidus with delayed diffuse supratentorial leukoencephalopathy with microhemorrhages without concurrent hypoxia or reported preceding symptoms suggestive of hypoxia. We suspect that these inflammatory mechanisms might be triggered by prior COVID-19 exposure/infection. Furthermore, the role of the cross-talk between inflammation and clinically mild or silent hypoxia linked to prior COVID-19 infection cannot be excluded. Awareness of these post–COVID-19 neurological sequelae and their potential pathophysiology among those with no known antecedent significant hypoxia are important for early recognition and treatment.

## Introduction

Globus pallidus are known for their high metabolic activity and demand linked to their richness in blood flow and mitochondrial density (1, 2). Thus, they are among the brain regions that are susceptible to pathological conditions associated with significant metabolic disruption (1). These conditions include hypoxia, metabolic abnormalities (e.g., hyperammonemia, hypoglycemia, hyperglycemia, and hypoxemia), toxic exposure (e.g., carbon monoxide, cyanide, and methanol), mitochondrial disorders (Leigh disease), neurodegenerative causes (e.g., neurodegeneration with brain iron accumulation, Creutzfeldt–Jakob disease), and rarely Central nervous system (CNS) inflammatory disorders (e.g., neuro-Bechet disease) (1). Recently, bilateral symmetric globus pallidus lesions have been reported in COVID-19 infection with co-morbid hypoxia (3). While post–COVID-19 acute necrotizing encephalopathy (ANE) is a rare post-infectious, acute neuroinflammatory disorder characterized by rapid neurological decline, seizures, reduced level of consciousness, and bilateral symmetrical neuroimaging abnormalities (4, 5), it is consistently associated with bilateral globus pallidus lesions and involves the thalami symmetrically.

Acute hypoxia and other toxic–metabolic causes are the dominant etiologies among those with isolated involvement of globus pallidus (6). Delayed hypoxic–ischemic leukoencephalopathy has also been shown to be a cause of encephalopathy in the setting of benzodiazepine, cocaine, heroin use, carbon monoxide poisoning, methanol poisoning, mitochondrial dysfunction, and COVID-19 (7–9). In these cases, an original insult that caused hypoxia caused a delayed and/or prolonged encephalopathy which was also marked by radiographic findings of symmetric T2-FLAIR hyperintensities in the central white matter and sparing of the internal capsule, periventricular region, cerebellum, and brainstem (8).

We report a case of rapidly progressive cognitive decline of subacute onset initially associated with isolated symmetric globus pallidus structural abnormalities on brain magnetic resonance imaging (MRI) followed by delayed diffuse leukoencephalopathy in a patient found to have clinically unrecognized, but resolved, antecedent COVID-19 infection without current or prior evidence of hypoxia, respiratory distress, critical illness, or toxic–metabolic abnormalities. This presentation, together with brain PET findings of mild temporal lobe hypermetabolism superimposed on diffuse cerebral hypometabolism, suggested post–COVID-19 dysregulation of immunity and inflammation. Immunotherapy was shown to be highly efficacious, further supporting the suspected diagnosis.

## Case description and diagnostic assessment

A 63-year-old female nurse in the medical intensive care unit with a medical history of hyperlipidemia and mitral valve regurgitation presented with a 3-week history of rapidly progressive cognitive decline and psychomotor slowing, preceded by several weeks of malaise and chills but without documented or reported fever, shortness of breath, or hypoxia. Of note, the patient received the Pfizer–BioNTech COVID-19 vaccine about 6 months prior to the onset of her symptoms. A CT head without contrast was performed 3 days prior to admission during an emergency department visit for the same symptoms, which showed bilateral basal ganglia hypo-intensities. At that time, the patient did not have any significant neurological deficits and chose to leave the hospital against medical advice before further workup could be completed. She presented again to the emergency department, and upon admission, the patient was afebrile with stable vital signs. There were no meningismus signs or skin rash. A neurological exam revealed severe global psychomotor slowing, minimal verbal output, significant impairment of comprehension and attention, motor paucity with the minimally increased tone of all extremities, and a slow shuffling gait. Laboratory studies showed positive COVID-19 spike antibodies at >250 U/mL [reference negative <0.79], positive nucleocapsid antibodies at 15.6 [reference negative <0.99], and negative nasal SARS-CoV-2 RT-PCR testing suggestive of prior COVID-19 infection, in addition to the previously known vaccination. Aside from a mild transaminitis that peaked on day 13 of hospitalization, the remaining basic serologies and immunoassays were largely unremarkable including white blood cell count, erythrocyte sedimentation reactivity, c-reactive protein, antinuclear antibodies, double-stranded DNA antibody, and a paraneoplastic antibody panel.

**Figure 1:**
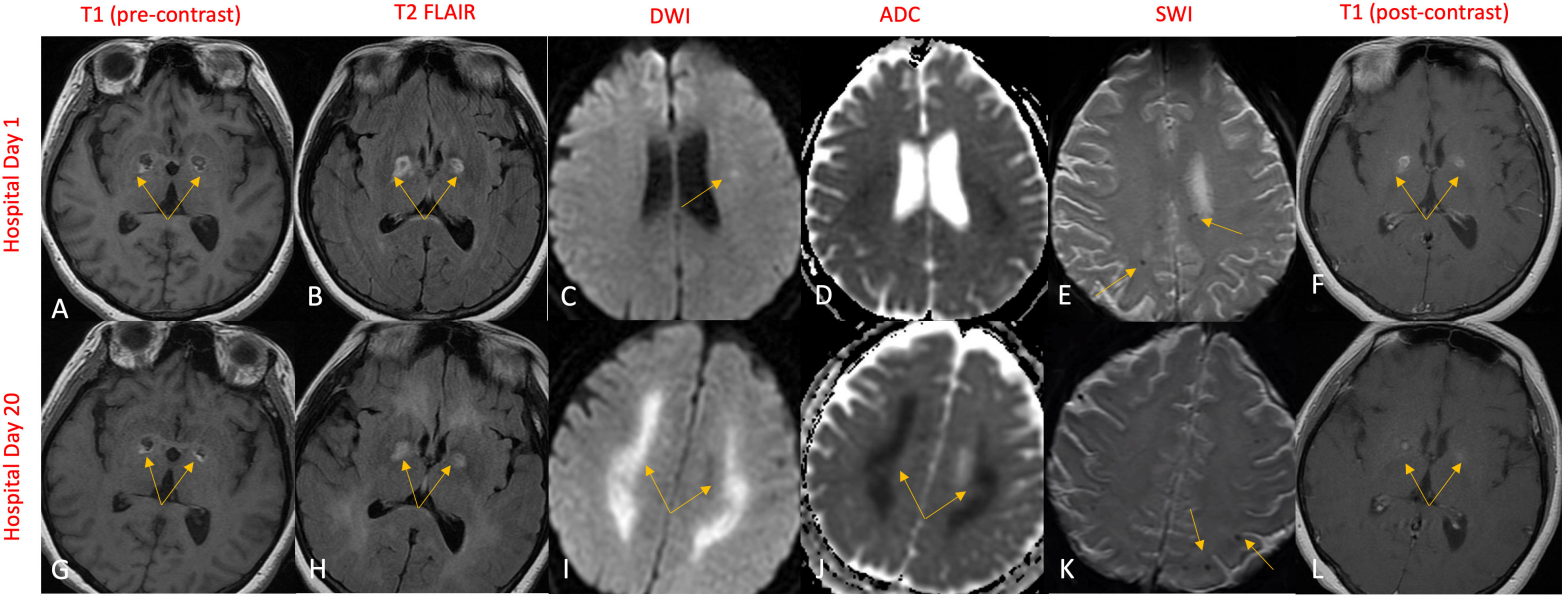
MR brain on hospital day 1 revealed symmetric bilateral globus pallidus lesions that exhibited T1 and T2 rim hyperintensities **(A, B)** and contrast enhancement **(F)** engulfing a T1 and T2 hypointense necrotic core **(A, B)**. Subtle diffusion restriction of the corona radiata was also demonstrated **(C, D)**, along with several punctate areas of susceptibility signal loss in the supratentorial white matter indicative of microhemorrhages **(E)**. Repeat imaging on hospital day 20 revealed significant progression in diffusion restriction **(I)** with ADC correlate **(J)** now extensively involving the supratentorial white matter. The globus pallidus lesions and microhemorrhages remained largely unchanged **(G, H, K, L)**.

**Figure 2:**
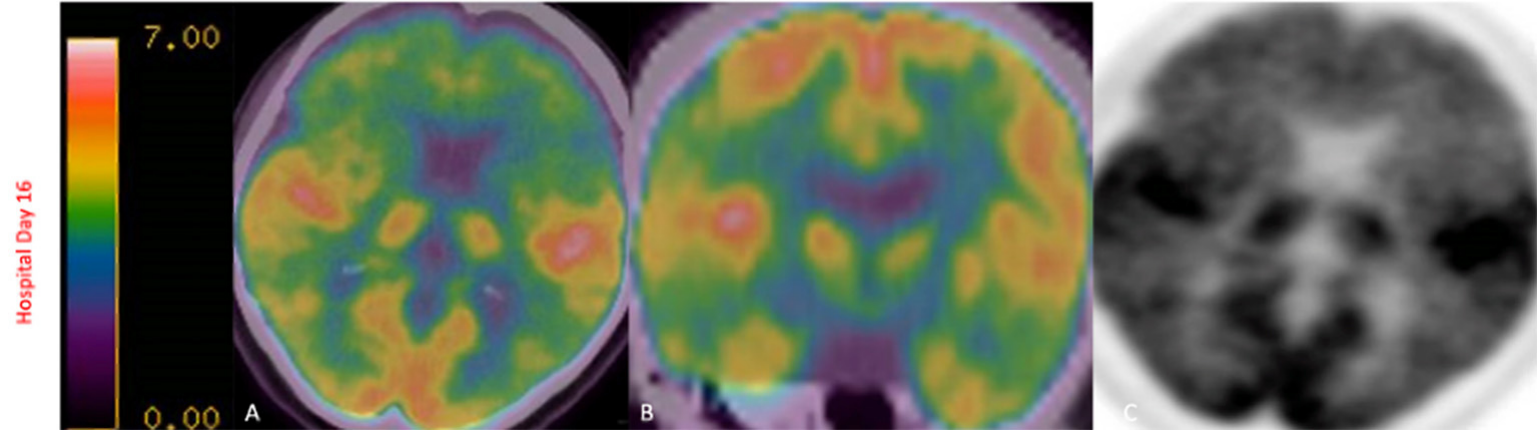
FDG-PET scan on hospital day 16 revealed decreased focal uptake in both globus pallidus, diffuse supratentorial hypometabolism with relatively increased tracer uptake in both anteromedial temporal lobes, including the hippocampal formations, parahippocampal gyrus, and amygdala, findings that have been described in limbic encephalitis **(A–C)**.

**Figure 3:**
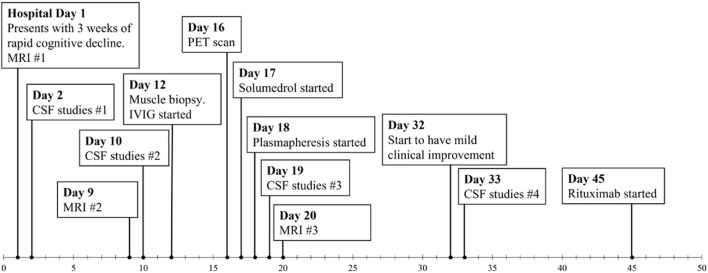
Timeline.

Gadolinium-enhanced brain MRI on hospital day 1 revealed bilateral symmetrical enhancing necrotic lesions of globus pallidus, which were hyperintense on T1- and T2-weighted imaging (Figures 1A,B,F). There was diffusion restriction in the left corona radiata (Figure 1C), together with diffuse foci of susceptibility loss throughout the supratentorial white matter (Figure 1E). CSF analysis on hospital day 2 revealed no pleocytosis, with normal protein and glucose levels (Table 1). CSF myeline basic protein (MBP) was not tested. Extended EEG monitoring for several days revealed generalized rhythmic delta activity without associated epileptiform abnormalities. Since the initial neuroradiographic abnormalities were highly suggestive of a toxic or metabolic etiology, extensive toxic–metabolic diagnostic workup was performed, including Hgb A1c, serum glucose, urea, liver function tests, bilirubin, vitamins B1, B6, B12, folate, thyroid panel, urine toxicology, ammonia, hepatitis panel, lead, arsenic, manganese, mercury, methyl alcohol, ceruloplasmin, copper, cyanide, iron, immunoglobulin assay, methemoglobin, carboxyhemoglobin, amino acid assay, serum, and CSF paraneoplastic antibody panel, which was negative. In addition, an extensive rheumatological panel was unremarkable, including double-stranded DNA Ab, smooth muscle Ab, JO-1 Ab, Smith Ab, anti-ribonuclear protein, c-ANCA, p-ANCA, myeloperoxidase Ab, proteinase 3 Ab, Sjogren Ab, rheumatoid factor, thyroperoxidase Ab, CCP antibody, centromere Ab, and scleroderma Ab. In addition, the neuromyelitis antibody was negative. Abnormal values included cadmium levels at 2.3 ug/L (normal 0–1.2 ug/L), ferritin at 416 ng/mL (normal 15–150 ng/mL), and ANA levels at 1:80 (normal <1:80) in a nucleolar pattern. An evaluation of interleukin levels revealed elevated IL-6 and IL-10. The patient continued to show a progressive cognitive decline, together with a reduced level of alertness. Repeat contrast MR brain on hospital day 9 was unchanged. Repeat CSF analysis on hospital day 10 revealed no pleocytosis, mildly elevated protein levels of 47 mg/dL (reference 15–45 mg/dL), and elevated MBP of 94.3 ng/mL (reference <4.7 ng/mL) (Table 1). Arginine infusion for 5 days, together with nutritional supplements including coenzyme Q, taurine, and B-vitamin, was administered for possible mitochondrial disorder awaiting muscle biopsy that was later revealed to be negative. After a reasonable exclusion of alternative toxic–metabolic etiologies and given the lack of respiratory symptoms or hypoxia during this hospitalization (although the possibility of clinically unrecognized or silent hypoxia prior to hospitalization could not be totally excluded), together with the progressive neurological decline, post–COVID-19 CNS inflammation and/or dysregulation of immunity were suspected. This was supported by the reported preceding history of poorly defined medical illness associated with positive serology for nucleocapsid antibodies, but negative nasal PCR. Thus, the patient was treated with intravenous immunoglobulins (IVIGs) for 5 days without significant improvement. A brain positron emission tomography (PET) scan was performed on hospital day 16 to better define the pathophysiology and extent of CNS involvement. It revealed decreased focal uptake in both globus pallidus and diffuse supratentorial cerebral hypometabolism, but relatively increased tracer uptake in both anteromedial temporal lobes, including the hippocampal formations, parahippocampal gyrus, and amygdala. The mild hypermetabolism of both anteromedial temporal lobes suggested possible inflammatory mechanisms (Figure 2), and the patient was accordingly treated with IV methylprednisolone 1 g/day for 7 days and plasmapheresis for seven sessions. Repeat CSF analysis on hospital day 19 showed no pleocytosis and relative improvement of MBP levels to 63.6 ng/mL (Table 1). Repeat contrast-enhanced brain MRI on hospital day 20 revealed new diffuse symmetric diffusion restricting lesions involving the supratentorial white matter with associated T2-FLAIR hyperintensity (Figure 1I). The globus pallidus lesions and microhemorrhages were unchanged (Figures 1G, H, L, K). Contrast-enhanced CT of the chest, abdomen, and pelvis to exclude paraneoplastic etiology was unremarkable. The pelvic ultrasound was negative. By hospital day 32, the patient was noted to have mild clinical improvement including improved attention and alertness, increased speech output, and the ability to speak in short phrases and follow basic commands. Repeat CSF analysis on hospital day 33 continued to reveal no pleocytosis with a steady decline of MBP to 35.3 ng/mL (Table 1). Given the observed clinical response following immunotherapies, rituximab was initiated on hospital day 45. She was discharged to rehab after a 52-day hospital stay. At the 6-month follow-up visit, she demonstrated significant improvement in all neurological domains with fluent speech, near complete recovery of comprehension abilities, and good recall of recent and remote events. An MRI performed at the 6-month follow-up showed a decrease in T2 hyperintensities. The chronicity of events is shown in Figure 3.

**Table 1:** CSF studies.

**Hospital day**	**Opening pressure (reference range 6–25 cmH2O)**	**Total nucleated cell count (reference range 0–5/uL)**	**Red blood cell count (reference range 0/uL)**	**Glucose (reference range 40–70 mg/dL)**	**Protein (reference range 15–45 mg/dL)**	**Myelin basic protein (reference range 0–4.7 ng/mL)**
Day 2	7	2	3	64	38	—
Day 10	17	<1	**7**	**74**	**47**	**94.3**
Day 19	13	3	**7**	**94**	33	**63.6**
Day 33	12	2	**94**	**76**	36	**35.3**

Bold values are significantly abnormal values.

## Discussion

We report a case of a patient that developed rapidly progressive cognitive and neurological decline initially associated with bilateral symmetric contrast-enhancing necrotic lesions of globus pallidus, restricted diffusion of the left corona radiata, and cerebral punctate hemorrhages that later progressed to profound supratentorial diffuse leukoencephalopathy. Two prior case reports found bilateral basal ganglia involvement in patients with COVID-19 infection: one found bilateral hypodensities on CT imaging suggestive of acute ischemic stroke but was unable to have any further imaging due to clinical deterioration and subsequent death (10), and the other found bilateral hyperdensities suggestive of a hemorrhagic event which was confirmed on MRI imaging (11). Our patient stands in contrast to these cases as the noted globus pallidus lesions do not appear to have a vascular etiology. Although similar neuroimaging abnormalities have previously been reported among those with severe toxic–metabolic derangements, mitochondrial encephalopathies, and certain neurodegenerative disorders (1, 2), after the exclusion of these relevant etiologies, we believe this case is suggestive of post–COVID-19 CNS inflammation and/or dysregulation of immunity and is unique for the following reasons: (1) The absence of a severe hypoxic insult; although preceding mild hypoxia not recognized by the patient and her family cannot be totally excluded, mild hypoxia alone is likely insufficient to result in such severe progressive structural abnormalities manifesting with rapid and profound global neurological decline as seen in cases of delayed hypoxic leukoencephalopathy from COVID-19 who had profound hypoxia requiring mechanical ventilation (9); (2) the history of preceding and poorly defined constitutional symptoms with negative nasal SARS-CoV-2 RT-PCR testing but with nucleocapsid antibodies and seropositivity on admission suggests a possible mechanistic link to antecedent COVID-19 infection (12). Notably, similar neuroimaging structural abnormalities, diffuse confluent T2-white matter hyperintensities with sparring of the U-fibers, and scattered microhemorrhages in subcortical white matter, have been previously reported in active COVID-19 infections with severe systemic manifestations, critical illness, or severe hypoxia (3, 13–16), but not in post–COVID-19 infections without these medical sequelae, such as in this case. Furthermore, in a study of 25 consecutive mechanically ventilated patients with COVID-19 infection, nearly half of the patients were found to have evidence of either diffuse leukoencephalopathy, microhemorrhages, or both (16). These findings differed significantly from a study conducted at the same institution of 254 consecutive patients—not limited to those who were critically ill or mechanically ventilated—which did not find a single case of diffuse leukoencephalopathy (12). These studies suggested that COVID-19-related leukoencephalopathy may be common in critically ill patients, but rare in less severe infections, and is etiologically related to severe hypoxemia and hypercytokinemia storm; and (3) the findings of structural preservation of oxygen-sensitive mesial temporal lobe structures with mild bilateral anteromedial temporal hypermetabolism, together with the clinically significant response to immune therapies, suggest a potential contributory role of post–COVID-19 CNS inflammation and/or dysregulation of immunity, particularly after a reasonable exclusion of alternative etiologies. This inflammatory role might become more etiologically relevant in the presence of possible antecedent unrecognized mild hypoxia, given the experimental data supporting bidirectional cross-talk between inflammation and hypoxia through several mechanisms, including hypoxia-inducible factor, nuclear factor-kappa B, and immune cells recruitment (17). In addition, the elevated interleukins 6 and 10 provide additional evidence of an immune-mediated response causing significant neuro-inflammation. Future studies exploring the potential role of the interplay between hypoxia and inflammation and immune signaling in the neurological sequelae during and after COVID-19 infection are needed.

Awareness of these post–COVID-19 neurological sequelae and their potential pathophysiology among those with no known antecedent significant hypoxia are important for early recognition and treatment with immune therapies to optimize the clinical outcomes and limit the neurological, cognitive, and behavioral deficits.

## Data availability statement

The raw data supporting the conclusions of this article will be made available by the authors, without undue reservation.

## Ethics statement

Ethical review and approval was not required for the study on human participants in accordance with the local legislation and institutional requirements. The patients/participants provided their written informed consent to participate in this study. Written informed consent was obtained from the individual(s) for the publication of any potentially identifiable images or data included in this article.

## Author contributions

AB, MK, YC, MA, AH, and SN contributed to conceptualization, literature search, writing, review, and editing. AB and SN verified the underlying data. All authors contributed to the article and approved the submitted version.
